# Supplementary material for: Benefits of Chlorella vulgaris against Cadmium Chloride-Induced Hepatic and Renal Toxicities via Restoring the Cellular Redox Homeostasis and Modulating Nrf2 and NF-KB Pathways in Male Rats
Source: Biomedicines. 2023 Aug 29;11(9):2414. doi: 10.3390/biomedicines11092414 (PMC10525457; doi:10.3390/biomedicines11092414)
Supplement: Supplementary file 1 [file biomedicines-11-02414-s001.zip › biomedicines-2483842-supplementary.pdf]

Supplementary Materials

Table S1. Primer sequences (5′-3′) used for the quantitative real-time qPCR.

Genes	Forward (5'-3')	References
NF-kB	F-GCT TTG CAA ACC TGG GAA TA<br>R-CAA GGT CAG AAT GCA CCA GA	[1]
HSP70	F-GCT GAC CAA GAT GAA GGA GAT<br>R-GCT GCG AGT CGT TGA AGT AG	[2]
Nrf2	F-ACA CAG CAT AGC CCA TCT CGT<br>R-ACC AAC CTG GAT GAG CGA CAC	[3]
HO-1	F-CTT CCC GAG CAT CGA CAA C<br>R-CTG TCA CCC TGT GCT TGA CC	[3]
β-actin	F- TCACTATCGGCAATGTGCGG<br>R- GCTCAGGAGGAGCAATGATG	[3]

References

1. Preuss, H.G.; Jarrell, S.T.; Scheckenbach, R.; Lieberman, S.; Anderson, R.A. Comparative effects of chromium, vanadium andgymnema sylvestre on sugar-induced blood pressure elevations in SHR. *J. Am. Coll. Nutr.* **1998**, *17*, 116–123.
2. Khalil, S.R.; Awad, A.; Mohammed, H.H.; Nassan, M.A. Imidacloprid insecticide exposure induces stress and disrupts glucose homeostasis inmale rats. *Environ. Toxicol. Pharmacol.* **2017**, *55*, 165–174.
3. Pakzad, M.; Fouladdel, S.; Nili-Ahmadabadi, A.; Pourkhalili, N.; Baeeri, M.; Azizi, E.; Sabzevari, O.; Ostad, S.N.; Abdollahi, M. Sublethal exposures of diazinon alters glucose homostasis in Wistar rats: biochemical and molecular evidences of oxidative stress in adipose tissues. *Pestic. Biochem. Physiol.* **2013**, *105*, 57–61.
